# Effects of exercise training and exercise plus acupuncture on chronic insomnia: a feasibility study

**DOI:** 10.5935/1984-0063.20220053

**Published:** 2022

**Authors:** Walkyria Silva Ferreira, Marcos Gonçalves Santana, Shawn D Youngstedt, Daniela Elias de Assis, Bernardo Pessoa de Assis, Daniela Pinto de Cerqueira, Marcia Carolina Mazzaro, Giselle Soares Passos

**Affiliations:** 1 Universidade Federal de Jataí, Programa de Pós-graduação em Ciências Aplicadas à Saúde - Jataí - GO - Brazil; 2 Universidade Federal de Jataí, Curso de Educação Física - Jataí - GO - Brazil; 3 Arizona State University, Edson College of Nursing and Health Innovation - Phoenix - AZ - United States

**Keywords:** Exercise, Sleep, Depression, Anxiety, Acupuncture, Sleep Initiation and Maintenance Disorders

## Abstract

**Objective:**

The aim of this study was to investigate the effects of exercise and exercise plus acupuncture on chronic insomnia.

**Material and Methods:**

suggest replacing with “effects of” no feasibility things are reported chronic insomnia were randomized to a 12-week treatment with exercise or exercise plus acupuncture. Exercise treatment included 50 minutes of moderate-intensity aerobic exercise (50% of reserve heart rate), on a treadmill, 3 times/wk. Exercise plus acupuncture treatment included the exercise protocol plus acupuncture once per week. Pre- and post-treatments measures included insomnia severity index (ISI), Pittsburgh sleep quality index (PSQI), polysomnography (PSG), 10 days-sleep diary, state-trait anxiety inventory, Beck depression inventory, quality of life (SF-36), and morning cortisol level.

**Results:**

No group by time interaction was found for insomnia severity, sleep, mood or quality of life. Significant time differences (*p*<0.05) were observed for ISI, PSQI, and some variables of sleep diary. Polysomnography data showed a decrease in rapid eye movement (REM) latency after the interventions. Significant time improvements were also observed for mood, anxiety, depression, and quality of life. A significant moderate correlation was found between changes in the ISI and morning cortisol level.

**Conclusion:**

There were no significant differences between treatments on insomnia severity, sleep, mood or quality of life. Exercise and exercise plus acupuncture were efficacious for decreasing insomnia severity to subthreshold insomnia. Greater reduction in morning cortisol was associated with a greater reduction on insomnia severity across both treatments.

## INTRODUCTION

Chronic insomnia is a sleep complaint that affects between 9 and 15% of the population worldwide and is associated with impairments in daytime functioning^1^, including reduced alertness, fatigue, dysphoria and other symptoms. Insomnia has been also associated with a 24h increase of adrenocorticotropic hormone and cortisol secretion, consistent with a disorder of central nervous system hyperarousal^2^. Hyperarousal during sleep and wakefulness have been hypothesized for chronic insomnia^3^.

Treatment based on drugs is the most usual, but advances in management of chronic insomnia have been proposed^4^. Combined techniques have been tested to potentiate the positive effects of drug or non-drug treatments^5^,^6^. Regular exercise has been a promising alternative treatment in the last couple of decades^7^,^8^. Positive effects after acute exercise^9^,^10^ and exercise training^11^-^14^ have been found for sleep, mood and quality of life of patients with chronic insomnia. Moderate-intensity aerobic exercise has been shown to improve sleep in indiviuals with chronic insomnia^9^,^12^-^14^. Acupuncture is one of the alternative therapies for insomnia and is widely used around the world^15^-^17^. There is evidence that it has had additive effects to the effects of drugs^17^. The association of exercise and acupuncture could result in additive improvements on sleep.

Some mechanisms support the effects of acupuncture and exercise on chronic insomnia. Studies reported acupuncture can improve sleep quality by suppression of central nervous system activity and increases in the content of gamma-amino butyric acid (GABA)^18^,^19^. Antidepressant effect and anxiety reduction are the most studied mechanisms to explain the effects of exercise on sleep^20^ and insomnia. An additive effect was hypothesized, analogous to additive effects of exercise and acupuncture, which have been found for pain^21^.

The primary aim of this study was to examine the effects of exercise training and exercise training plus acupuncture on chronic insomnia. A secondary aim was to examine the effects of the intervention on sleep quality, self-reported sleep, mood, quality of life, and morning cortisol level and to examine correlations of sleep changes with cortisol changes.

## MATERIAL AND METHODS

### Participants and screening

Participants were recruited through newspaper advertisements and online media. Prospective participants contacted the researchers and were initially screened in a phone interview. Inclusion criteria were: (a) aged ≥25 and <60 years; (b) diagnosis of chronic insomnia based on a combination of criteria from the diagnostic and statistical manual of mental disorders DSM-V and the International Classification of Sleep Disorders (ICSD-3). These criteria were further operationalized as difficulties initiating and/or maintaining sleep at least three nights per week; insomnia duration longer than three months; and significant distress or impairment of daytime functioning. Exclusion criteria were: (a) use of psychoactive drugs; (b) history of psychiatric diseases; (c) shift work; (d) regular exercise (>1day/week) and/or acupuncture treatment for insomnia in the last 6 months; (e) body mass index (BMI) >30.

Prospective participants who passed a phone screen were invited to the “Sleep Clinic” for further orientation. During the visit, the prospective participants signed a written informed consent form approved by the ethics committee. A medical screening (conducted by a neurologist expert in sleep disorders) included clinical diagnosis of insomnia and coexistence of major depression. The cardiologist extensively interviewed each patient about cardiovascular risks and performed a resting EKG as well as EKG assessment during the exercise.

A baseline polysomnography excluded participants with an apnea-hypopnea index (AHI) >15 or periodic leg movement index (PLMI) >15. Patients using sleep medications no more than twice weekly were enrolled after they withdrew from the medications for at least two weeks. Ethical approval for all experimental measures was granted by the University Human Research Ethics Committee (Universidade Federal de Goiás, #1.998.334) and conformed principles outlined in the declaration of Helsinki (clinical trial registration # NCT03171519).

### Treatments

Following screening, participants were randomized to one of two 12-week treatments.

### Moderate-intensity aerobic exercise training

The exercise sessions were performed three times per week (Monday, Wednesday and Friday) on a treadmill (Embreex, 567 GT1), for 50 minutes, at an intensity relative to 50% of the reserve heart rate (HRR) ±5bpm, during each session over 12 weeks. The training was between 11 a.m. and 2 p.m. (which is the traditional lunch/siesta time in Brazil). All sessions were preceded by five minutes of warm-up, followed by stretching of the upper and lower limbs and followed by five minutes of active recovery. HRR was re-calculated at the end of the 4^th^ and 8^th^ weeks by the formula ([**(max HR - resting HR) × %Intensity] + resting HR).** HRmax was calculated by traditional formula “220 minus age” and resting HR was registered by a Polar FT1.

### Exercise plus acupuncture

The treatment protocol included the association of aerobic exercise and acupuncture. Aerobic exercise was performed following the moderate-intensity aerobic exercise protocol. The acupuncture treatment was performed by a trained acupuncturist (physical therapist expert in sleep) once a week, on Tuesdays or Thursdays, between 11:00 and 14:00h. In the acupuncture session, based on traditional Chinese medicine (TCM) meridian theory, the following acupoints were manually stimulated: Baihui (Governor Vase - VG20), located in the center-vertical line of the head, 7 tsun from the posterior edge of the hair; YinTang (Extra point 15), located between the eyebrows; Anmian (Extra point 30), located in a depression posterior to the base of the ear, approximately 1 tsun; Shenmen (Heart-C7): located radially to the pisiform bone and the tendon of the flexor carpi ulnaris muscle; Neiguan (Pericardium-PC6): located between the tendons of the radial flexor carpi and long palmar muscles, 2 tsun above the wrist line; Sanyinjiao (Spleen-Pancreas-BP6): on the posteromedial border of the tibia, 3 tsun above the medial malleolus. Indeed, the points located on the outer ear Shenmen, heart, occiput, subcortex^16^. All selected points were registered in a meta-analysis evaluated the effects of acupuncture on chronic insomnia and were effective improving symptoms of insomnia^15^,^16^.

For the acupuncture session, threaded acupuncture needles, sterile, disposable, with diameter (0.25x15Mm) were used. The insertion of the needles was performed at 90º in relation to the body and ear surface, with light intensity, performing rotation movements (clockwise and counterclockwise) for approximately 15 seconds at each point or until reaching de-qi, characterized by a sensation of numbness, distension or tingling at the site of the needle radiating along the corresponding meridian which is considered an essential feature of acupuncture therapy according to the TCM^22^. The needle remained at each point for 50 minutes (90)^23^. Before the procedure, the stitches were sanitized, as well as the applicator’s hand.

### Design and procedures

**Figure 1:**
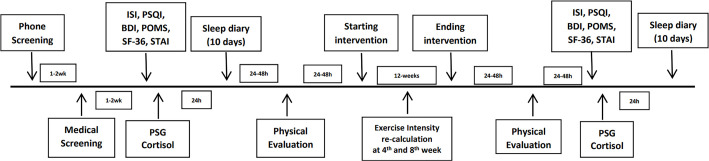
Timeline of study design.

Notes: ISI = Insomnia severity index; PSQI = Pittsburgh sleep quality index; BDI = Beck depression inventory; POMS = Profile of mood states; SF-36 = Short form-36; STAI = State-trait anxiety inventory; PSG = Polysomnography.

The design included two evaluations: baseline (pre-intervention) and after 12-week intervention (post-intervention). All evaluations were conducted according to the design (Figure 1).

### Measures

#### Insomnia severity

Insomnia severity index (ISI) assessed insomnia-related complaints. It is a short and easy self-applied scale with 7 items scored from 0 to 4, with a total score varying from 0 to 28. The total score is interpreted as follows: absence of insomnia (0-7); sub-threshold insomnia (8-14); moderate insomnia (15-21); and severe insomnia (22-28)^24^.

#### Subjective sleep quality

Pittsburgh sleep quality index (PSQI) was used to assess sleep quality over the previous 4 weeks in the pre and post-intervention. Sleep onset latency, sleep duration and sleep efficiency (ratio between sleep duration and total bedtime by 100) were obtained. Global score >5 indicate poor sleep quality^25^.

#### Sleep diary

Participants kept daily sleep diaries during a 10-days baseline period and after 12-weeks treatments. The primary dependent variables derived from the diaries were sleep onset latency (SOL), wake time after sleep onset (WASO), total sleep time (TST), sleep efficiency (SE; ratio of sleep time to the time spent in bed), and sleep debt (SD). Sleep debt was calculated by the difference between the hours obtained in the first question of sleep diary “how many hours do you need sleep to be restored in the following morning? And the mean of TST obtained during 10 days. The sleep diary is a standard assessment instrument in insomnia research^26^, which allows for prospectively monitoring sleep patterns over extended periods in the patient’s home.

#### Polysomnography (PSG)

At a Sleep Clinic (NEUROCOR, Jataí, GO, Brazil) the PSG was performed using the ICELERA (*i blue*, version 1.1.39) device at different periods of 30-second windows classified as awake, sleep stages N1, N2, and N3 (non-rapid eye movement - NREM), and REM (rapid eye movement) sleep according to the criteria standardized by Iber et al. (2007)^27^ Four EEG leads (C3-A2, C4-A1, Fz-A1, and O1-A1), 2 EOG channels (C3), 2 EMG channels (submental and legs), and1 ECG lead (modified D2) were recorded. The recording started according to patient’s habitual bedtime and finished at 7 a.m. Sleep variables analyzed were: total sleep time (TST), sleep efficiency (SE; ratio between total sleep time and total time of recording multiplied by 100), sleep onset latency (SOL), REM latency (LREM), wake time after sleep onset (WASO), arousals, apnea hypopnea index (AHI), periodic leg movements (PLM), and percentage of sleep stages. The analysis of the events in the polysomnography was carried out by two investigators who used international criteria and were blind to the grouping of the volunteers.

#### State-trait anxiety, depression, mood and quality of life

Anxiety was evaluated by the STAI scales. They encompasses 20 items and provide a one dimensional measurement of trait and state anxiety^28^. The range of scores for each subtest is 20-80, with higher scores indicating greater anxiety. Depression symptoms was scored by the Beck depression inventory (BDI)^29^. It is a 21-question multiple-choice self-report inventory with answers that comprise scores range from 0 to 3 (absent, mildly, moderately, and severely). The minimum score is 0 and the maximum score is 63. The BDI cutoffs are: 0-9 minor or no depression symptoms; 10-18 mild depression symptoms; 19-29 moderate depression symptoms; and 30-63 severe depression symptoms. Profile of mood states (POMS) was the instrument used to evaluate mood states. It has 65 items and 6 domains: tension-anxiety, depression, anger-hostility, vigour-activity, fatigue, and confusion-bewilderment. The total mood disturbance score is derived by subtracting the vigour-activity score from the sum of scores from the other subscales^30^. Medical Outcomes Study - SF-36 questionnaire was used to assess the quality of life. It includes 8 components: physical functioning, physical role, body pain, general health, vitality, social functioning, emotional role, and mental health. All scores ranged from 0 to 100, with a higher score indicating better quality of life^31^.

#### Morning cortisol level

Sample blood was collected at 08:00 ± 1h, at baseline and post-treatment at the “Sleep Clinic” following the PSG, before the participants ate, drank, or brushed their teeth. Serum cortisol was analyzed by chemiluminescence.

#### Physical evaluation

To assess changes in body composition of patients (body mass, fat%, and fat-free mass), a body composition monitor (Omron, HBF-514) was used. Height was assessed using a flexible tape measure (Sanny Medical, SN-4010) to calculate the body mass index (body mass/height^2^).

#### Data analysis

TIBCO Statistica^TM^ software (version 13.5) was used for analysis. The primary outcome variable (ISI score) was analyzed group x time (two groups and two times) repeated-measures analysis of variance (ANOVA). Changes in secondary outcomes (sleep, mood, anxiety, depression, quality of life, and morning cortisol level) were evaluated in an identical fashion to ISI scores. Hedges *g* was used to verify the effect sizes between groups and the time effect was also verified by Hedges *g* paired effect size. According to the convention, effect sizes of 0.20-0.39, 0.40-0.79, and >0.8 are considered small, medium, and large, respectively. Pearson’s correlations were conducted to assess whether changes (delta%) on insomnia severity (ISI score) and sleep variables were associated with changes in anxiety, depression, mood, quality of life, and morning cortisol level. Data are presented as mean (S.D.). Significance level was at *p*<0.05.

## RESULTS

**Figure 2:**
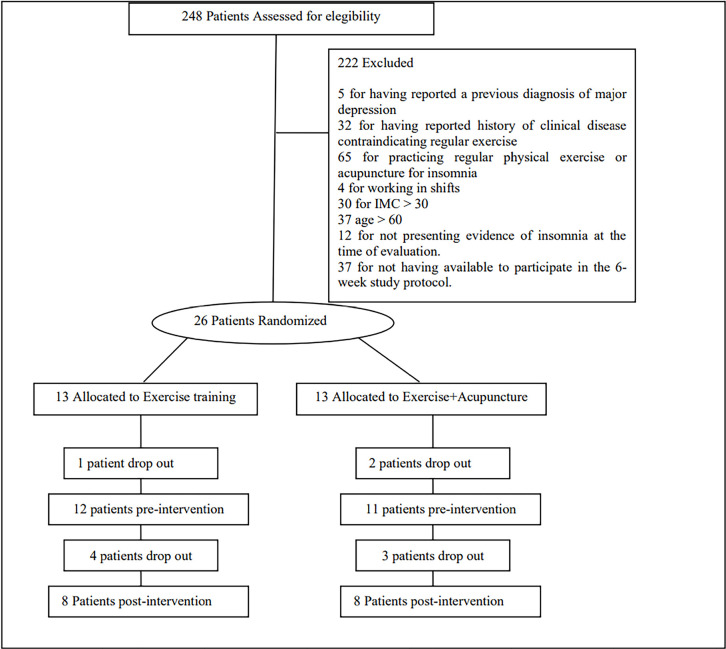
Participant flowchart.

Two hundred and forty-eight people were interested in taking part in the study and contacted the researchers by telephone or email. Of these, 222 did not meet the inclusion criteria and were excluded (Figure 2).

Twenty-six participants were randomized to the exercise training group (n=13) or exercise plus acupuncture group (n=13). However, 3 men and 3 women withdrew from the study during the baseline period before they began exercise training. Thus, the exercise protocol began with 12 participants in the exercise group and 11 participants in the exercise plus acupuncture group. However, during the protocol, 4 participants in the exercise group and 3 participants in the exercise plus acupuncture dropped out of the trial for personal reasons not explained.

The final sample size was 8 participants in the exercise training group and 8 participants in the exercise plus acupuncture group. All of these 16 participants successfully completed the 12-week intervention. When they missed one session, they were assigned to replace this training session any other weekday in the same week. Thus, all patients were able to complete the 12-week protocol.

The mean age was 46.6 (8.3) and 43.2 (10.7) years and BMI was 26.6 (1.8) and 25.4 (1.0) for exercise training and exercise plus acupuncture, respectively. Gender (M/F) was 2/6 and 1/7 for exercise training and exercise plus acupuncture, respectively. The mean duration of insomnia was 10.4 (11.1) and 6.2 (9.2) years for exercise training and exercise plus acupuncture, respectively. There were no significant baseline differences between the treatments for the results (*p*>0.05).

No group by time interaction was found for insomnia severity, sleep, mood and quality of life. Significant time differences were observed. Decrease for ISI (F_1, 14_=31.69, *p*<0.001), and PSQI (F_1, 14_=6.34, *p*=0.02), and some variables on the sleep diary: WASO (F_1, 14_=4.85, *p*=.04), sleep debt (F_1, 14_=11.09, *p*=0.005). Increase on TST (F_1, 14_=8.18, *p*=0.01) and a trend toward improvement in sleep efficiency (F_1, 14_=4.37, *p*=0.05). These data are reported in Table 1. Polysomnography data showed decrease in REM latency (F_1, 14_=4.91, *p*=0.04) after both interventions (Table 2).

**Table 1:** Insomnia severity index (ISI), Pittsburgh sleep quality index (PSQI), and sleep variables obtained from sleep diary (mean of 10 days).

						ANOVA (p)		
	**Group**	**Pre-intervention**	**Post-intervention**	**Hedges g paired**	**Hedges g**	**Group**	**Time**	**Group x Time**
**ISI (0-28)**	EXERCISE	18.1 ± 4.6	11.9 ± 1.9	-1.66	0.13	ns	<0.01	ns
	EXE+ACU	19.0 ± 4.1	12.1 ± 5.1	-1.41				
**PSQI (0 -21)**	EXERCISE	13.4 ± 2.8	9.3 ± 3.7	-1.18	0.33	ns	0.02	ns
	EXE+ACU	11.9 ± 3.6	9.5 ± 2.4	-0.74				
**Total sleep time (h)**	EXERCISE	4.3 ± 1.1	5.1 ± 0.7	0.82	0.04	ns	0.01	ns
	EXE+ACU	4.3 ± 0.9	5.1 ± 1.0	0.79				
**Total time in bed (h)**	EXERCISE	7.8 ± 0.7	7.9 ± 0.5	0.15	0.57	ns	ns	ns
	EXE+ACU	7.8 ± 1.1	8.3 ± 0.7	0.51				
**Sleep onset latency (min)**	EXERCISE	38.4 ± 7.4	39.7 ± 19.8	0.08	0.21	ns	ns	ns
	EXE+ACU	39.4 ± 21.5	46.0 ± 24.7	0.27				
**Wake after sleep onset (min)**	EXERCISE	123.9 ± 68.4	88.2 ± 41.6	-0.60	0.11	ns	0.04	ns
	EXE+ACU	149.3 ± 84.5	105.9 ± 47.0	-0.60				
**Sleep efficiency (%)**	EXERCISE	55.6 ± 13.3	65.0 ± 9.7	0.76	0.21	ns	ns	ns
	EXE+ACU	57.7 ± 13.9	64.1 ± 14.9	0.42				
**Sleep debt (h)**	EXERCISE	2.6 ± 1.6	1.4 ± 1.2	-0.80	0.26	ns	<0.01	ns
	EXE+ACU	3.4 ± 1.2	2.5 ± 1.6	-0.60				

**Notes:** Repeated-measures analysis of variance (ANOVA); EXERCISE = Exercise training group (n=8); EXE+ACU = Exercise plus acupuncture group (n=8); ns = Not significant; Data are displayed as mean (standard deviation); *p*<0.05 taken to indicate significance.

**Table 2:** Sleep variables obtained from polysomnography.

						ANOVA (p)		
	**Group**	**Pre-intervention**	**Post-intervention**	**Hedges g paired**	**Hedges g**	**Group**	**Time**	**Group x time**
**Total sleep time (min)**	EXERCISE	439.9 ± 82.7	444.3 ± 82.7	0.05	0.87	ns	ns	ns
	EXE+ACU	451.4 ± 63.5	404.0 ± 60.0	-0.72				
**Sleep onset latency (min)**	EXERCISE	8.9 ± 5.1	4.8 ± 3.6	-0.88	0.18	ns	ns	ns
	EXE+ACU	7.5 ± 2.6	6.3 ± 10.1	-0.15				
**REM latency (min)**	EXERCISE	97.8 ± 41.9	81.1 ± 9.3	-0.52	0.52	<0.01	0.04	ns
	EXE+ACU	183.5 ± 47.0	138.1 ± 49.4	-0.89				
**Sleep efficiency (%)**	EXERCISE	85.1 ± 7.2	88.5 ± 6.4	0.47	0.79	ns	ns	ns
	EXE+ACU	87.7 ± 7.7	85.7 ± 8.4	-0.23				
**Wake after sleep onset (min)**	EXERCISE	66.1 ± 28.5	52.8 ± 28.6	-0.44	0.95	ns	ns	ns
	EXE+ACU	52.8 ± 30.0	66.7 ± 38.7	0.38				
**Stage 1 (%)**	EXERCISE	6.1 ± 3.3	6.0 ± 2.7	-0.03	0.31	ns	ns	ns
	EXE+ACU	6.0 ± 4.6	7.0 ± 5.2	0.19				
**Stage 2 (%)**	EXERCISE	50.8 ± 7.8	51.3 ± 9.4	-0.05	0.32	ns	ns	ns
	EXE+ACU	58.3 ± 8.1	55.9 ± 8.6	-0.27				
**Stage 3 (%)**	EXERCISE	22.9 ± 8.9	22.9 ± 9.1	0.00	0.42	ns	ns	ns
	EXE+ACU	21.5 ± 3.8	24.2 ± 9.3	0.36				
**REM (%)**	EXERCISE	20.2 ± 6.2	19.8 ± 5.0	-0.07	0.24	ns	ns	ns
	EXE+ACU	14.2 ± 3.9	12.8 ± 4.3	-0.32				
**AHI (events/h)**	EXERCISE	8.2 ± 9.4	10.3 ± 11.2	0.19	0.81	ns	ns	ns
	EXE+ACU	4.8 ± 4.1	3.3 ± 3.7	-0.36				

**Notes:** Repeated-measures analysis of variance (ANOVA); ns = Not significant; EXERCISE = Exercise training group (n=8); EXE+ACU = Exercise plus acupuncture group (n=8); AHI = Apnea-hypopnea index; Data are displayed as mean (standard deviation); *p*<0.05 taken to indicate significance.

Significant time differences were also observed in mood, anxiety and depression symptoms and in the quality of life of patients. Decreases in POMS subscales-tension/anxiety (F_1,14_=7.78, *p*=0.01), anger/hostility (F_1,14_=8.00, *p*=0.01), fatigue (F_1,14_=12.61, *p*=0.003), vigour/activity (F_1,14_=6.81, *p*=.02), confusion/bewilderment (F_1,14_=15.85, *p*=0.001), total mood disturbance (F_1,14_=16.44, *p*=.001), and a trend toward decreases in depression (F_1,14_=4.37, *p*=0.05) are reported in the Table 3. Decrease in BDI (F_1,14_=18.36, *p*<0.001) and STAI-trait (F_1,14_=19.15, *p*<0.001) and STAI-state (F_1,14_=15.10, *p*=0.001) are also reported in the Table 3. Improvements on the quality of life components: physical role (F_1,14_=5.27, *p*=0.04), general health (F_1,14_=10.88, *p*=0.005) vitality (F_1,14_=7.37, *p*=0.02), emotional role (F_1,14_=5.76, *p*=0.03) are described in the Table 4.

**Table 3:** Clinical symptoms of depression, anxiety, and mood states.

						ANOVA (p)		
	**Group**	**Pre-intervention**	**Post-intervention**	**Hedges g paired**	**Hedges g**	**Group**	**Time**	**Group x Time**
**BECK**	EXERCISE	15.2 ± 6.3	9.8 ± 5.1	-0.89	0.22	ns	<0.01	ns
**(0-63)**	EXE+ACU	19.2 ± 6.4	12.5 ± 8.4	-0.84				
**STAI**	EXERCISE							
**(20-80)**	EXE+ACU							
**State**	EXERCISE	45.4 ± 6.5	40.5 ± 11.2	-0.50	0.69	ns	<0.01	ns
	EXE+ACU	49.8 ± 10.2	38.6 ± 5.9	-1.27				
**Trait**	EXERCISE	49.6 ± 9.1	42.4 ± 9.2	-0.74	0.07	ns	<0.01	ns
	EXE+ACU	48.9 ± 6.8	43.0 ± 9.5	-0.67				
**POMS (score)**								
**Tension-anxiety**	EXERCISE	10.9 ± 7.2	4.1 ± 6.4	-0.94	0.07	ns	0.01	ns
	EXE+ACU	13.0 ± 7.2	6.9 ± 9.7	-0.67				
**Depression**	EXERCISE	15.0 ± 12.3	8.9 ± 5.5	-0.60	0.69	ns	ns	ns
	EXE+ACU	10.1 ± 6.6	8.9 ± 10.6	-0.13				
**Anger-hostility**	EXERCISE	12.3 ± 10.7	6.0 ± 6.9	-0.66	0.05	ns	0.01	ns
	EXE+ACU	11.5 ± 7.3	5.8 ± 6.3	-0.79				
**Vigour-activity**	EXERCISE	14.4 ± 4.6	15.9 ± 6.1	0.26	0.65	ns	0.02	ns
	EXE+ACU	15.6 ± 4.9	20.1 ± 4.6	0.89				
**Fatigue**	EXERCISE	10.7 ± 3.1	6.0 ± 4.8	-1.10	0.18	ns	<0.01	ns
	EXE+ACU	11.6 ± 7.0	7.7 ± 6.4	-0.55				
**Confusion-bewilderment**	EXERCISE	4.4 ± 3.6	1.3 ± 3.1	-0.87	0.17	ns	<0.01	ns
	EXE+ACU	3.9 ± 5.2	1.3 ± 4.0	-0.53				
**Total mood disturbance**	EXERCISE	38.9 ± 32.2	10.3 ± 25.7	-0.93	0.14	ns	<0.01	ns
	EXE+ACU	34.0 ± 24.3	9.1 ± 27.7	-0.90				

Notes: Repeated-measures analysis of variance (ANOVA); ns = Not significant; EXERCISE = Exercise training group (n=8); EXE+ACU = Exercise plus acupuncture group (n=8); POMS = Profile of mood states; Data are displayed as mean (standard deviation); *p*<0.05 taken to indicate significance.

**Table 4:** Quality of life evaluated by the SF-36 questionnaire.

						ANOVA (p)		
	**Group**	**Pre-intervention**	**Post-intervention**	**Hedges *g* paired**	**Hedges *g***	**Group**	**Time**	**Group x Time**
**Physical functioning (0-100)**	EXERCISE	86.9 ± 14.1	86.9 ± 15.8	0.00	0.69	ns	ns	ns
	EXE+ACU	76.3 ± 14.1	88.1 ± 16.2	0.73				
**Role-physical (0-100)**	EXERCISE	78.1 ± 36.4	93.8 ± 11.6	0.55	0.38	ns	0.04	ns
	EXE+ACU	46.9 ± 41.1	78.1 ± 31.2	0.81				
**Body pain (0-100)**	EXERCISE	62.3 ± 27.3	64.4 ± 22.1	0.08	0.44	ns	ns	ns
	EXE+ACU	40.0 ± 13.2	51.4 ± 21.0	0.61				
**General health perception (0-100)**	EXERCISE	71.5 ± 21.5	82.8 ± 18.7	0.53	0.71	ns	<0.01	ns
	EXE+ACU	56.5 ± 28.1	85.0 ± 18.1	1.14				
**Vitality (0-100)**	EXERCISE	41.0 ± 15.0	61.9 ± 16.2	1.27	0.28	ns	0.02	ns
	EXE+ACU	44.4 ± 21.8	58.1 ± 27.1	0.53				
**Social functioning (0-100)**	EXERCISE	70.3 ± 29.1	68.8 ± 29.9	-0.05	0.61	ns	ns	ns
	EXE+ACU	51.6 ± 29.5	68.8 ± 25.0	0.60				
**Role-emotional (0-100)**	EXERCISE	87.5 ± 24.8	91.7 ± 15.4	0.19	0.34	0.03	ns	ns
	EXE+ACU	50.0 ± 43.6	70.8 ± 45.2	0.44				
**Mental health (0-100)**	EXERCISE	53.5 ± 17.9	56.4 ± 27.1	0.12	0.60	ns	ns	ns
	EXE+ACU	45.5 ± 27.1	64.0 ± 20.4	0.73				

Notes: Repeated-measures analysis of variance (ANOVA); ns = Not significant; EXERCISE = Exercise training group (n=8); EXE+ACU = Exercise plus acupuncture group (n=8); Data are displayed as mean (standard deviation); *p*<0.05 taken to indicate significance.

**Figure 3:**
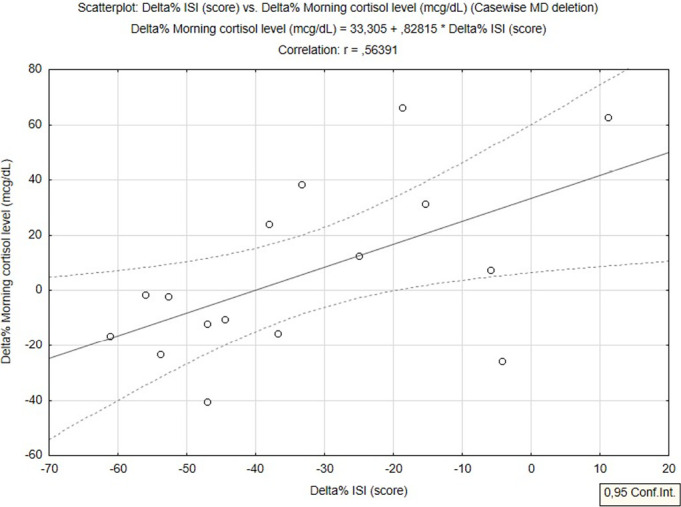
Correlation between changes in the ISI and morning cortisol level considering combined groups.

A significant interaction effect (time x group) for morning cortisol level (F_1,14_=4.89, *p*=0.04) is reported in Table 5. A moderate correlation was found between changes in the ISI and morning cortisol level (r=0.56, *p*<0.05) considering the combined groups (Figure 3).

**Table 5:** Physical evaluation and morning cortisol level.

						ANOVA (p)		
	**Group**	**Pre-intervention**	**Post-intervention**	**Hedges *g* paired**	**Hedges *g***	**Group**	**Time**	**Group x Time**
**Body mass (kg)**	EXERCISE	69.8 ± 9.0	69.6 ± 9.0	-0.02	0.25	ns	ns	ns
	EXE+ACU	69.7 ± 10.7	70.3 ± 13.8	0.05				
**% FAT**	EXERCISE	40.0 ± 5.6	37.6 ± 5.4	-0.41	0.72	ns	ns	ns
	EXE+ACU	36.5 ± 7.6	39.7 ± 10.0	0.34				
**FFM (kg)**	EXERCISE	25.1 ± 3.1	25.9 ± 4.1	0.21	0.33	ns	ns	ns
	EXE+ACU	27.5 ± 5.0	27.0 ± 4.9	-0.09				
**Morning cortisol level (mcg/dL)**	EXERCISE	13.6 ± 5.6	15.5 ± 3.9	0.53	1.10	ns	ns	0.04
	EXE+ACU	15.5 ± 5.4	13.3 ± 3.1	-0.47				

Notes: Repeated-measures analysis of variance (ANOVA); ns = Not significant; EXERCISE = Exercise training group (n=8); EXE+ACU = Exercise plus acupuncture group (n=8); FFM = Free fat mass; Data are displayed as mean (standard deviation); *p*<0.05 taken to indicate significance.

No significant correlations were found between changes in insomnia severity or sleep improvements and mood, depression, anxiety or quality of life.

## DISCUSSION

In this study, we have identified no additional beneficial effects of acupuncture on sleep beyond those found for exercise training in individuals with chronic insomnia, although we have used the acupoints suggested in the previous meta-analysis, similar frequency, and duration of treatment sessions. There were no significant differences between the groups for insomnia severity, sleep, mood or quality of life. Time effect was observed for ISI, and a large magnitude time effects was found for ISI across both treatments in the exercise training and exercise plus acupuncture. Sleep data revealed time effects for PSQI, sleep diary-TST, sleep diary-WASO, sleep diary-sleep debt, and PSG-REM latency and medium to large magnitude was observed for these variables. Improvements on sleep are consistent with previously reported responses to similar protocols of moderate-intensity aerobic exercise^12^, ^14^ and resistance exercise^11^. In the present study the protocol included 12-weeks and previous studied have included 16-weeks to 6 months. However, we found similar effects on insomnia and sleep after 12-weeks.

Previous meta-analysis evaluating acupuncture for chronic insomnia have described positive effects on PSQI and sleep duration^17^,^18^. Yeung et al. (2012)^16^ in a meta-analyses of the moderate-quality randomized control trials found that acupressure as monotherapy fared marginally better than sham control. Studies that compared auricular acupressure and sham control showed equivocal results. It was also reported that acupressure, reflexology, or auricular acupressure as monotherapy or combined with routine care was significantly more efficacious than routine care or no treatment. The authors concluded that considering the methodological limitations of the studies and equivocal results, the current evidence does not allow a clear conclusion on the benefits of acupressure, reflexology, and auricular acupressure for insomnia.

Mood improvements were observed in both groups after treatments. Though not significantly different, the magnitude of exercise plus acupuncture changes in anxiety-state, POMS-vigour/activity was larger (*g*=-1.27; *g*=0.89, respectively). Quality of life data also revealed time effect for both groups, and medium to large paired effect sizes for role-physical, general health perception and vitality. Though not significantly different, the magnitude of exercise plus acupuncture changes in role-physical and general health perception was larger (*g*=0.81; *g*=1.14, respectively). Similar results on mood and quality of life after exercise on patients with chronic insomnia was also reported previously^11^,^12^,^14^.

A significant interaction effect was observed for morning cortisol level. After 12-week moderate-intensity aerobic exercise alone the participants had a significant increased morning cortisol level, whereas after exercise plus acupuncture a significant decrease was observed. A previous study has showed decrease on morning cortisol level after 4-months moderate intensity aerobic exercise^13^. Investigation of cortisol in the morning in trials that evaluate the effects of acupuncture on chronic insomnia have been proposed^32^. However, the evidences about the relationship between chronic insomnia and cortisol level are mixed^3^. Previous studies have shown evidences for low cortisol in the morning in people with insomnia^33^,^34^. Some authors have suggested that insomniacs have sustained arousal and activation of their stress system, an around-the-clock activation of the HPA axis^35^. However, there is evidence that higher levels of morning cortisol are associated with worse sleep^33^.

Contrary to other literature, in the present study there was a significant moderate positive correlation between changes in ISI and morning cortisol level. Higher ISI was associated with higher morning cortisol level considering all patients investigated in this study (combined groups).

This a feasibility study. The results should be interpreted with caution. The small sample size is the first limitation in this study. However, the paired effect sizes showed moderate to large magnitude, similar of previous studies that evaluated the effects of exercise on chronic insomnia. Time in bed varied by >90 min between pre and post for some subjects. The absence of significant additional effects of acupuncture on chronic insomnia could be explained by only one time/week session, which could not be enough to addict positive effects on chronic insomnia. However, previous studies have studied protocols with acupuncture once week as adjuvant therapy^34^ or primary treatment^36^ and found positive results on sleep. The effects of acupuncture might have also been limited by the standardized approach, which we chose for this initial study. Traditional acupuncture uses an individualized approach to sites on the body for administering acupuncture.

In conclusion, there were no significant differences between treatments on insomnia severity, sleep, mood or quality of life. Exercise and exercise plus acupuncture were feasible for decrease insomnia severity to subthreshold insomnia. Greater reduction in morning cortisol was associated with a greater reduction on insomnia severity across both treatments.
